# Event-related brain responses to emotional words, pictures, and faces – a cross-domain comparison

**DOI:** 10.3389/fpsyg.2014.01106

**Published:** 2014-10-06

**Authors:** Mareike Bayer, Annekathrin Schacht

**Affiliations:** Courant Research Centre Text Structures, University of GöttingenGöttingen Germany

**Keywords:** emotion, language, pictures, facial expressions of emotions, event-related brain potentials (ERPs), domain specificity, positivity bias, negativity bias

## Abstract

Emotion effects in event-related brain potentials (ERPs) have previously been reported for a range of visual stimuli, including emotional words, pictures, and facial expressions. Still, little is known about the actual comparability of emotion effects across these stimulus classes. The present study aimed to fill this gap by investigating emotion effects in response to words, pictures, and facial expressions using a blocked within-subject design. Furthermore, ratings of stimulus arousal and valence were collected from an independent sample of participants. Modulations of early posterior negativity (EPN) and late positive complex (LPC) were visible for all stimulus domains, but showed clear differences, particularly in valence processing. While emotion effects were limited to positive stimuli for words, they were predominant for negative stimuli in pictures and facial expressions. These findings corroborate the notion of a positivity offset for words and a negativity bias for pictures and facial expressions, which was assumed to be caused by generally lower arousal levels of written language. Interestingly, however, these assumed differences were not confirmed by arousal ratings. Instead, words were rated as overall more positive than pictures and facial expressions. Taken together, the present results point toward systematic differences in the processing of written words and pictorial stimuli of emotional content, not only in terms of a valence bias evident in ERPs, but also concerning their emotional evaluation captured by ratings of stimulus valence and arousal.

## INTRODUCTION

A smiling face, a sad headline on a newspaper: emotional stimuli seem to easily attract our attention in everyday live. And in fact, a wealth of evidence has established the preference of emotional information across a variety of stimuli and has traced it back to the level of neuronal processing. It is the aim of the present study to compare emotion effects in event-related brain potentials (ERPs) between the three stimulus domains most often encountered within the visual modality, namely for pictures of emotional scenes or objects, facial expressions of emotions, and written words of emotional content.

Numerous studies using ERPs reported evidence for preferential processing of emotional pictures (Cuthbert et al., 2000; Schupp et al., 2000; for review, see Olofsson et al., 2008), facial expressions of emotions (Schupp et al., 2004b; Rellecke et al., 2012), and emotional words (e.g., Herbert et al., 2008; Schacht and Sommer, 2009a,b; Bayer et al., 2012b). These studies suggest general similarities between stimulus domains in ERP effects elicited by emotional content. This seems noteworthy considering the vast differences between stimulus domains in general: In the first place, these differences concern physical stimulus properties (e.g., words as symbolic stimuli compared to pictorial stimuli, i.e., pictures and faces); furthermore, they extend to theoretical considerations. As an example, it was recently proposed that pictures of objects or scenes might provide *direct* affective information, while facial expressions of emotion only constitute *indirect* affective information since they primarily convey the emotion of the person depicted (Walla and Panksepp, 2013). Despite these differences, however, a number of ERP components were frequently shown to be similarly elicited or modulated by emotional stimulus content. At around 100 ms after stimulus onset, the P1 component reflects the perceptual encoding of visual input. Modulations for emotional as compared to neutral stimuli were reported for words (e.g., Scott et al., 2009; Bayer et al., 2012b; Keuper et al., 2012), pictures (Delplanque et al., 2004), and facial expressions (e.g., Rellecke et al., 2012). Subsequently, enhanced sensory processing of emotional stimuli is indexed by the so-called early posterior negativity (EPN, e.g., Junghöfer et al., 2001; Schupp et al., 2004a; Kissler et al., 2007; Herbert et al., 2008; Bayer et al., 2012a). Although the EPN occurs with comparable scalp distributions, its latency clearly differs between stimulus domains: For pictures and faces, the EPN usually starts around 150 ms after stimulus onset, whereas its onset for emotional words has been located at a post-lexical processing stage at around 250 ms after stimulus onset (e.g., Palazova et al., 2011). Finally, starting from ∼300–400 ms after stimulus onset, an enhanced parietal positivity for emotional stimuli was suggested to reflect higher-order stimulus evaluation [late positive complex (LPC), e.g., Cuthbert et al., 2000; Schupp et al., 2000; Herbert et al., 2008; Schacht and Sommer, 2009a; Bayer et al., 2012a]. Although LPC amplitudes have been reported for both emotional words, pictures, and facial expressions, the scalp topography of the LPC was reported to differ between stimulus domains, thus indicating the involvement of at least partially different brain structures in the elaborate processing of emotional stimulus content (Schacht and Sommer, 2009a).

Despite the notable similarities in the emotional processing mentioned above, only little is known about the actual comparability of emotion effects between stimulus domains in terms of effect strength, automaticity, or possible valence biases. Clear conclusions are complicated for two reasons. First, the comparability *across* studies is severely limited by specificities of experimental designs and procedures, stimulus materials, and task demands; all of which may influence emotion effects in ERPs (Fischler and Bradley, 2006; Schacht and Sommer, 2009b). Second, only a relatively small number of studies has realized direct, that is *within*-subject, comparisons of emotion effects between stimulus domains; to the best of our knowledge, none of them employing all three visual domains mentioned above at the same time and under comparable task demands.

Despite these limitations, previous research has generated evidence for two diverging accounts about differences in emotion effects between stimulus domains. First, it was suggested that words might be generally less capable of triggering emotion effects than pictures and facial expressions. This difference was explained with a supposedly lower arousal level of symbolic stimuli, i.e., words, in general, which might thus elicit weaker arousal responses (De Houwer and Hermans, 1994; Hinojosa et al., 2009). In a study by Hinojosa et al. (2009), emotion effects for words in an intactness decision were limited to the LPC time window from 350 to 425 ms. In contrast, emotional pictures additionally elicited effects in reaction times (RTs) and in the earlier EPN component^1^. Similar results were reported for emotional facial expressions. In a study by Rellecke et al. (2011), employing superficial face-word decisions, both emotional facial expressions and emotional words elicited emotion effects already at early latencies between 50 and 100 ms after stimulus onset, but later EPN effects were limited to facial stimuli. On theoretical grounds, these results are in line with the notion of a cascaded response to stimuli according to their biological relevance and thus to their possible impact on the well-being of the observer (Lang et al., 1997). This assumption was corroborated by reduced emotion effects in facial muscle activity for words as compared to pictures and sounds (Larsen et al., 2003) and in the activity of the autonomous nervous system (for a discussion, see Bayer et al., 2011).

In contrast to these results, a number of recent studies reported similar activation patterns for emotional content of pictures, faces, and words, at least concerning the activity of the central nervous system. In an ERP study by Schacht and Sommer (2009a), both emotional facial expressions and emotional words elicited emotion effects in EPN and LPC amplitudes, albeit differing in time course, and, in case of the LPC, in scalp distributions. Similarly, Schlochtermeier et al. (2013), reported evidence for comparable emotion-related brain activity for emotional pictures and words in a fMRI study, while taking into account the differences of visual complexity between stimulus domains. Finally, both pictograms and words elicited similar modulations of the P300 component in an ERP study; anterior modulations in the LPC window were even more pronounced for words compared to pictograms (Tempel et al., 2013). Thus, it is conceivable that stimulus domains may not generally differ in their capacity to trigger emotion-related brain activity.

A further domain-specificity in emotion processing becomes evident when considering differential effects of emotional valence. Here, a large number of findings suggest the existence of valence biases, which differ between domains. For written words, a bias for positive stimuli has often been reported, which was evident not only in ERPs (e.g., Herbert et al., 2006; Kissler et al., 2009; Bayer et al., 2012b), but also in RTs (e.g., Schacht and Sommer, 2009b) and amygdala activity (Herbert et al., 2009). In the case of pictures, however, negative stimuli seem to attract preferential processing, resulting in augmented emotion effects for negative as compared to neutral or positive stimuli (Carretié et al., 2001; Smith et al., 2003; Delplanque et al., 2004). Finally, regarding emotional facial expressions, results suggest a facilitated processing of threatening faces as conveyed by angry or fearful facial expressions (Schupp et al., 2004b; Pourtois and Vuilleumier, 2006; Rellecke et al., 2012).

As in the case of domain comparisons (reporting generally reduced emotion effects in the verbal domain), these differences were again related to an assumed lower arousal level of written words (Herbert et al., 2006). Findings were interpreted in the framework of a theory proposed by Cacioppo and Gardner (1999) suggesting the existence of a preference of positively valenced stimuli at relatively low arousal levels (*positivity offset*) and a *negativity bias*, that is, a preferential processing of negative information at high arousal levels.

In summary, two major differences in the processing of emotional content between written words and pictorial stimuli (including pictures and facial expressions) arose from previous research. First, words in general were reported to be less capable of triggering emotion effects in ERPs (although a number of studies reported similar activation patterns). Second, a positive valence bias was frequently shown within the verbal domain, whereas a preference for negative content was reported for pictures and facial expressions. In both cases, these differences were supposed to originate from a generally lower arousal level of written words in comparison to pictures or facial expressions.

The present study had two major objectives. First, it aimed to investigate possible differences in emotion effects in ERPs between the three stimulus domains in a within-subject design. More precisely, it sought to answer the question whether (i) effects of emotional content would be reduced or absent in response to words as compared to pictures or facial expressions; or (ii) there would be a positive valence bias for words and a bias for negative valence for pictures and facial expressions. Both findings were reported in previous literature and were often supposed to result from lower arousal values for words compared to pictorial stimulus domains. Therefore, the second aim of this study was to provide empirical evidence for this theoretical assumption by collecting valence and arousal ratings for all experimental stimuli using an independent sample of participants.

Since several emotion effects have been shown to differentially depend on task demands (e.g., Schacht and Sommer, 2009b), the same task – a silent reading/passive viewing paradigm with occasional 1-back recognition memory tests – and experimental design was employed in order to achieve maximal comparability between stimulus domains. Above that, we decided to present stimuli in their most “naturalistic” form, thus accepting differences in physical features between stimulus domains like colorfulness, size, and complexity, in order to allow for results that are representative for each stimulus domain.

## MATERIALS AND METHODS

### PARTICIPANTS

Data was collected from 25 native German speakers; one data set was excluded from the analyses due to the participant’s left-handedness. The remaining 24 participants (mean age = 25.4 years, SD = 4.9) were right-handed (according to Oldfield, 1971), had normal or corrected-to-normal vision, and no phobias or other psychiatric or neurological disorders according to self-report. Participants received course credits or 20 Euros for participation. The study was designed according to the Declaration of Helsinki and was approved by the local institutional review board.

### STIMULI

Stimulus materials consisted of 72 faces, pictures, and words, each. Within each stimulus domain, the stimulus set contained 24 positive, 24 negative and 24 neutral stimuli.

Words were selected from the Berlin Affective Word List Reloaded (Vo et al., 2009); only nouns were included. Stimulus categories were controlled regarding word frequency (Baayen et al., 1995), word length (numbers of letters and syllables) and imageability ratings, all *F*s(2,69) < 1; for stimulus characteristics, see **Table 1**. Emotion categories differed significantly in their valence ratings as expected, *F*(2,69) = 1403.45, *p* < 0.001. Regarding stimulus arousal, positive and negative words were rated as significantly more arousing than neutral words, *F*s(1,46) > 100.86, *p*s < 0.001, but did not differ from each other, *F*(1,46) = 1.43, *p* = 0.714.

**Table 1:** Stimulus characteristics for words, pictures, and faces.

	Positive	Neutral	Negative
**Words**
Valence (-3 to 3)	2.1 (0.2)	0.3 (0.3)	-2.1 (0.3)
Valence (-2 to 2)	1.4 (0.1)	0.1 (0.2)	-1.3 (0.3)
Arousal (1 to 5)	3.3 (0.7)	1.9 (0.2)	3.5 (0.5)
Arousal (0 to 4)	2.3 (0.7)	0.9 (0.2)	2.5 (0.5)
Imageability (1 to 7)	5.5 (0.8)	5.6 (0.4)	5.5 (0.6)
Frequency (Ftot/1 mil)	27.7 (31.9)	24.6 (29.2)	24.8 (20.5)
Number of letters	6.3 (2.0)	6.3 (1.2)	6.4 (2.1)
**Pictures**
Valence (1 to 9)	7.3 (0.6)	5.1 (0.3)	2.9 (0.7)
Valence (-2 to 2)	1.2 (0.3)	0.0 (0.1)	-1.1 (0.3)
Arousal (1 to 9)	5.5 (0.9)	3.1 (0.5)	5.4 (0.7)
Arousal (0 to 4)	2.5 (0.5)	1.1 (0.3)	2.4 (0.4)
No. depicting people	12	7	10
Visual complexity	577.3 (111.4)	554.6 (175.1)	556.3 (187.2)
**Faces**
Valence (1 to 5)	4.5 (0.2)	2.9 (0.2)	1.3 (0.3)
Valence (-2 to 2)	1.5 (0.2)	-0.1 (0.2)	-1.7 (0.3)

*Additionally to listing valence and arousal ratings on the original rating scales, rating values were transformed to the scales used in the post-experimental ratings in order to allow for comparability between rating values.*

Picture stimuli were chosen from the IAPS database (Lang et al., 2008). As for word stimuli, emotion categories differed significantly in their valence ratings, *F*(2,69) = 446.57, *p* < 0.001. Positive and negative pictures were matched for arousal, *F*(1,46) < 1, but were significantly more arousing than neutral pictures, *F*s(1,46) > 151.07, *p*s < 0.001. Emotion categories did not significantly differ in their luminance, apparent contrast, and physical complexity as measured by JPEG file size, all *F*s(2,69) < 1, or in the number of pictures depicting humans, *F*(2,69) = 1.08, *p* = 0.344.

Face stimuli consisted of portraits of 72 different persons with happy, neutral, or angry facial expressions (*n* = 24 per category, 12 female). Faces were chosen from previous studies by Rellecke et al. (2012). Valence ratings confirmed that angry faces were perceived as more unpleasant than happy and neutral faces, and that happy faces were rated as more pleasant than neutral and angry faces, all *F*s(1,46) > 540.99, *p*s < 0.001. A rectangular gray mask with an ellipsoid aperture was added to the portraits in order to display solely the facial area.

### PROCEDURE

Before the start of the experiment, participants signed informed consent and provided demographic information. Stimuli were presented at the center of a computer screen positioned at a distance of 60 cm from the participant. At the beginning of each trial, a mask was presented for 1 s; corresponding to stimulus domain, the mask consisted of a scrambled word, picture, or face. Following this mask, stimuli were presented for 3 s. Words, pictures, and faces were presented in separate blocks; the order of blocks was counterbalanced. Within blocks, stimuli were presented twice in randomized order. After 10% of trials, stimuli were followed by a 1-back task in order to ensure participant’s attention to the stimuli. During these test trials, a stimulus was presented within a green frame, and participants indicated by button press whether the presented stimulus was identical to the preceding stimulus or not. Importantly, the position of test trials was randomized and thus unpredictable to the participant. Furthermore, all test trials were excluded from analyses. Words were presented in Arial font at font size 28 and spanned a mean visual angle of 2.4^∘^ × 0.9^∘^. Pictures had a size of 512 × 384 pixels, corresponding to a visual angle of 15.4^∘^ × 10.8^∘^; faces were presented at a size of ∼300 × 350 pixels, resulting in a visual angle of 8.6^∘^ × 11.4^∘^.

### DATA ACQUISITION

The EEG was recorded from 61 electrodes placed in an electrode cap according to the extended 10–20 system (Pivik et al., 1993); four electrodes placed at the outer canthi and below both eyes were used to record electro-oculograms. Signals were recorded with a sampling rate of 500 Hz and amplified with a bandpass filter of 0.032–70 Hz. Electrode impedance was kept below 5 kΩ. Electrodes were referenced to the left mastoid; oﬄine, data was re-referenced to average reference. Blinks were corrected using Surrogate Multiple Source Eye Correction implemented in Besa (Brain Electric Source Analysis, MEGIS Software GmbH). Epochs containing artifacts, i.e., amplitudes exceeding –100 or +100 μV or voltage steps larger than 50 μV, were discarded, resulting in the elimination of 1.5% of trials. Overall number of discarded trials per condition (domain by emotion) ranged between 13 and 21 and did not differ between conditions, as indicated by a repeated-measures ANOVA, all *F*s < 1.33. Continuous data was segmented into segments of 1100 ms, starting 100 ms prior to stimulus onset, and referred to a 100 ms pre-stimulus baseline.

### DATA ANALYSIS

**FIGURE 1:**
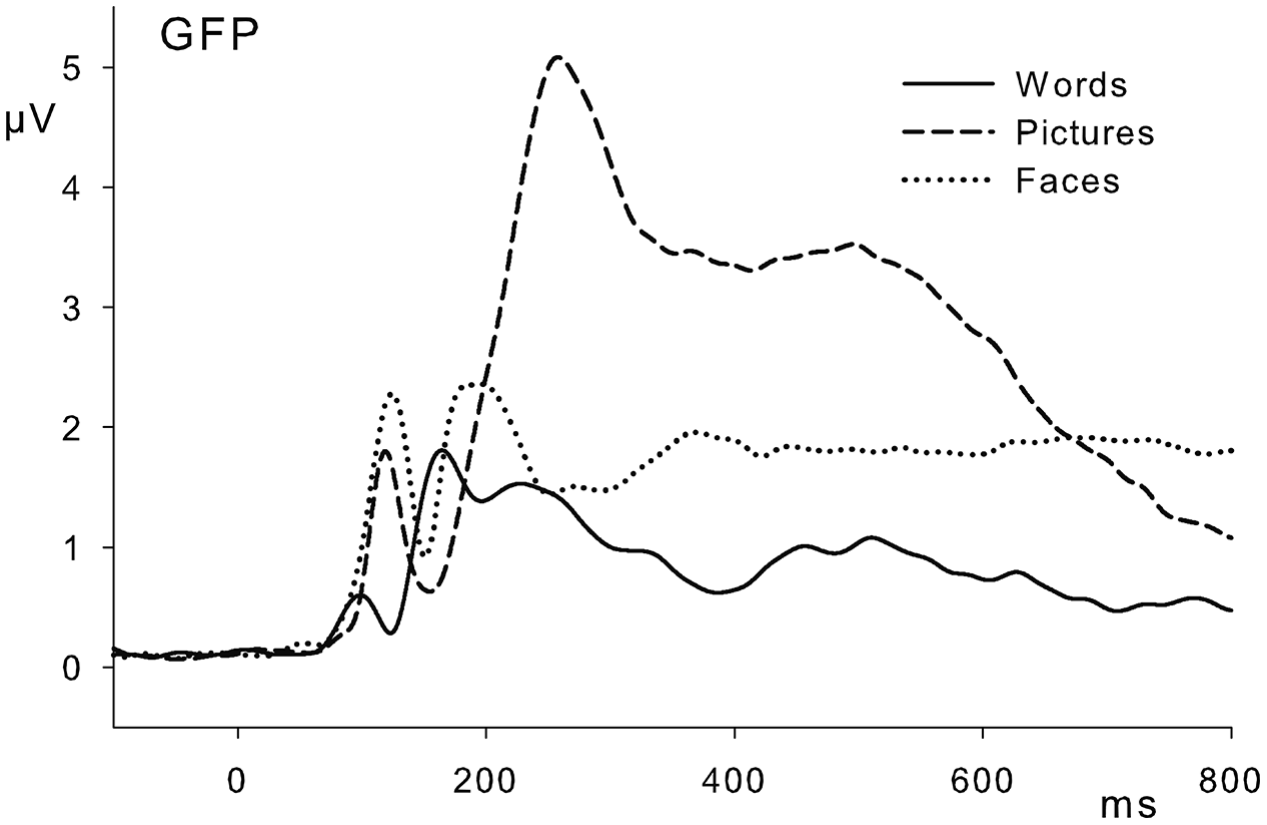
**Global field power (GFP) waveforms for words, pictures, and faces, averaged over emotion categories**.

Behavioral performance in the 1-back task was analyzed by a repeated-measures ANOVA including the factors domain (words, pictures, faces) and trial type (repeated stimulus, new stimulus). P1 amplitudes were determined by an automated peak-detection algorithm as maximal positive deflection between 50 and 150 ms after stimulus onset at occipital electrodes PO9, PO7, PO8, and PO10; electrode PO8 was used as reference channel. Modulations of the EPN were assessed as mean ERP amplitudes at a group of posterior electrodes (TP9, TP10, P9, P7, P8, P10, PO9, PO10, Iz); the LPC was quantified at a group of centro-parietal electrodes (CP1, CPz, CP2, P3, Pz, P4, PO3, POz, PO4). Since stimulus domains did show considerable differences in their general processing [see **Figure 1** for global field power (GFP) amplitudes], and in order to account for domain-related difference in the time course of emotion effects (Schacht and Sommer, 2009a), time windows for the analyses of EPN and LPC within each stimulus domain were determined by visual inspection of grand mean waveforms. The EPN was analyzed between 250 and 400 ms for words, between 180 and 250 ms for pictures, and between 170 and 300 ms for faces. For the LPC, time windows for analyses ranged from 500 to 650 ms (words), from 400 to 800 ms (pictures), and from 400 to 600 ms (faces). In the EPN time windows, we additionally analyzed anterior activations at electrode locations AFz, F3, Fz, F4, FC1, FCz, FC2. Within each stimulus domain, the influence of emotional content on P1, EPN, anterior positivity and LPC amplitudes was analyzed by repeated-measures ANOVAs including the factors emotion (positive, neutral, negative) and electrode (see above for specific electrode numbers and locations per region of interest); only significant main effects of emotion will be reported. In order to specify the onsets of EPN effects in each stimulus domain, onset analyses were performed on significant *post hoc* comparisons. To this aim, we applied running *t*-tests on grand averages of ERP differences between emotion conditions; in order to prevent spurious results, only activations with a minimum length of 10 consecutive significant data points were considered. Degrees of freedom in ANOVAs were adjusted using Huynh–Feldt corrections. Results will be reported with uncorrected degrees of freedom, but corrected *p*-values. Within *post hoc* tests, Bonferroni-corrections were applied to *p*-values; all significant and marginally significant results (<0.1) are reported.

### POST-EXPERIMENTAL RATING OF EMOTIONAL VALENCE AND AROUSAL

All stimuli used in the main experiment were rated for emotional valence and arousal by an independent sample of 67 participants (mean age = 24.1, SD = 3.4; 48 female) by using a computerized version of the self-assessment manikin (Bradley and Lang, 1994). Like in the main experiment, stimuli were present block-wise for each domain, in randomized order within each block; the order of blocks was counterbalanced. Furthermore, the sequence of ratings (valence and arousal) was counterbalanced. Ratings were aggregated over conditions and analyzed by ANOVAs including the factors emotion (positive, negative, neutral) and stimulus domain (words, pictures, faces). Alpha levels in *post hoc* comparisons were Bonferroni-corrected. For interactions between emotion and stimulus domain, only emotion comparisons across stimulus domains will be reported.

## RESULTS

### BEHAVIORAL DATA

Performance in the 1-back task was at 96.87%. An ANOVA on percentage of correct classifications yielded no significant differences between stimulus domains (words, pictures, faces) or trial type (repeated vs. new stimulus).

### EVENT-RELATED BRAIN POTENTIALS

#### Words

**FIGURE 2:**
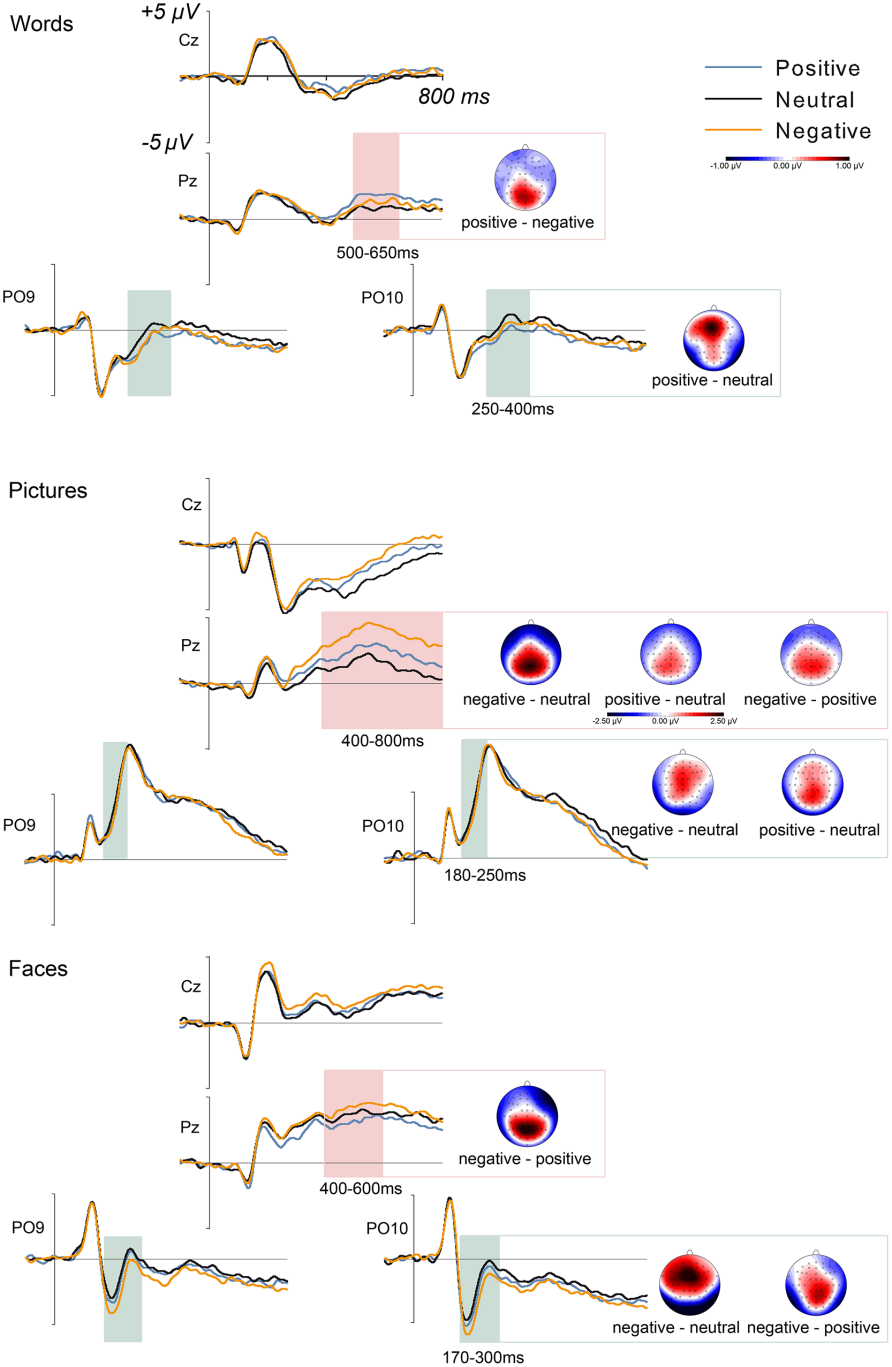
**Grand mean waveforms for positive, neutral, and negative words, pictures, and faces at selected electrode locations.** Highlighted areas show time windows of analyses for EPN and LPC in the respective stimulus domain. Scalp distributions of significant emotion effects are depicted as differences between indicated emotion categories; time windows correspond to the highlighted areas on the left side. The voltage scale of -1 to 1 μV applies to all topographies but the LPC for pictures, where the corresponding scale is depicted underneath the scalp distributions.

No emotion effects were visible in P1 peak amplitudes, *F*(2,46) < 1. In the EPN time window, ANOVAs revealed a significant main effect of emotion, *F*(2,46) = 6.48, $\eta_{p}^{2}$ = 0.220, reflecting larger amplitudes of the EPN for positive compared to neutral words, *F*(1,23) = 13.81, $\eta_{p}^{2}$ = 0.375; the onset of this effect was located at 250 ms. Analyses of the anterior positivity in the EPN time window showed a main effect of emotion, *F*(2,46) = 4.85, *p* < 0.05, $\eta_{p}^{2}$ = 0.174, reflecting larger amplitudes for positive than for neutral words, *F*(2,46) = 9.19, $\eta_{p}^{2}$ = 0.285. Furthermore, there was a main effect of emotion in the LPC time window, *F*(2,46) = 4.14, *p* < 0.05, $\eta_{p}^{2}$ = 0.152, which was based on a more pronounced positivity at centro-parietal electrodes for positive compared to negative words, *F*(1,23) = 7.43, *p* < 0.05, $\eta_{p}^{2}$ = 0.244, and, as a trend, for positive versus neutral words, *F*(1,24) = 5.17, *p* = 0.075, $\eta_{p}^{2}$ = 0.200. ERP results are depicted in **Figure 2**, for an overview see **Table 2**.

**Table 2:** Overview of emotion effects in EPN and LPC, including anterior positivities in the EPN time window.

	Words	Faces	Pictures
EPN	positive > neutral	negative > positive, neutral	negative, positive > neutral
Anterior positivity	positive > neutral	negative, positive > neutral	n.s.
LPC	positive > negative	negative > positive	negative > positive > neutral

*The table shows significant results of post-tests.*

#### Pictures

Analyses of P1 amplitudes revealed no significant effects of emotion, *F*(2,46) = 1.44, *p* = 0.247. For the EPN, ANOVAs revealed a main effect of emotion, *F*(2,46) = 5.53, *p* < 0.05, $\eta_{p}^{2}$ = 0.194. This effect was based on larger EPN amplitudes for negative relative to neutral pictures, *F*(1,23) = 9.22, *p* < 0.05, $\eta_{p}^{2}$ = 0.286, with an onset at 174 ms, and for positive compared to neutral pictures, *F*(1,23) = 7.20, *p* < 0.05, $\eta_{p}^{2}$ = 0.238, which started at 194 ms. At anterior electrodes, analyses revealed no significant activations, *F*(2,46) = 1.89, *p* = 0.162. A pronounced effect of emotion was observed in the LPC, *F*(2,46) = 5.98, *p* < 0.001, $\eta_{p}^{2}$ = 0.610. This effect was based on larger LPC amplitudes for negative pictures compared to both neutral pictures, *F*(1,23) = 53.89, *p* < 0.001, $\eta_{p}^{2}$ = 0.701, and positive pictures, *F*(1,23) = 24.47, *p* < 0.001, $\eta_{p}^{2}$ = 0.516. Furthermore, positive pictures elicited larger LPC amplitudes than neutral pictures, *F*(1,23) = 17.85, *p* < 0.001, $\eta_{p}^{2}$ = 0.437.

#### Faces

As for words and pictures, there were no emotion effects in P1 amplitudes, *F*(2,46) < 1. A main effect of emotion occurred for the EPN, *F*(2,46) = 15.87, *p* < 0.001, $\eta_{p}^{2}$ = 408; angry faces elicited larger EPN amplitudes relative to both positive faces, *F*(1,23) = 18.1, *p* < 0.001, $\eta_{p}^{2}$ = 0.440, and neutral faces, *F*(1,23) = 24.57, *p* < 0.001, $\eta_{p}^{2}$ = 0.516. The onsets of these effects were located at 176 ms (negative vs. positive) and at 168 ms (negative vs. neutral). In the same time window, a significant emotion effect was evident at anterior sites, *F*(2,46) = 15.78, *p* < 0.001, $\eta_{p}^{2}$ = 0.407, reflecting larger positive amplitudes for both negative and positive faces compared to neutral faces, *F*(1,23) = 24.87, *p* < 0.001, $\eta_{p}^{2}$ = 520, and *F*(1,23) = 21.32, *p* < 0.001, $\eta_{p}^{2}$ = 0.480, respectively. In the LPC time window, analyses showed a main effect of emotion, *F*(2,46) = 7.50, $\eta_{p}^{2}$ = 0.246, reflecting larger LPC amplitudes for negative relative to positive faces, *F*(1,23) = 15.57, *p* < 0.01, $\eta_{p}^{2}$ = 0.404.

### RATINGS OF EMOTIONAL VALENCE AND AROUSAL

#### Arousal

**FIGURE 3:**
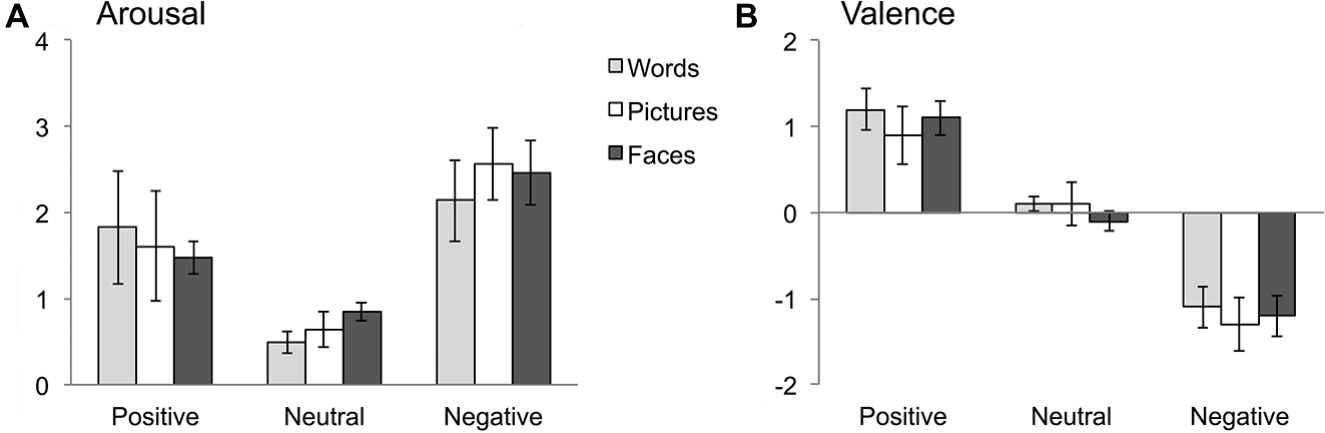
**Results of post-experimental ratings of stimulus arousal **(A)** and valence **(B)**.** Error bars indicate standard deviations.

Arousal ratings showed no main effect of stimulus domain, *F*(2,207) = 1.829, *p* = 0.163. There was a main effect of emotion category, *F*(2,207) = 329.11, *p* < 0.001, $\eta_{p}^{2}$ = 0.761, indicating that positive and negative stimuli were rated as more arousing than neutral stimuli; additionally, negative stimuli received higher arousal ratings than positive stimuli, all *p*s < 0.001. An interaction of emotion and stimulus domain, *F*(4,207) = 7.26, *p* < 0.001, $\eta_{p}^{2}$ = 0.123, indicated that differences for emotion categories across stimulus domains were limited to negative stimuli, where pictures received higher arousal ratings than words, *p* < 0.05. For rating results, see **Table 3** and **Figure 3**.

**Table 3:** Results of post-experimental ratings for words, pictures, and faces.

	Positive	Neutral	Negative
**Words**
Valence (-2 to 2)	1.2 (0.2)	0.1 (0.1)	-1.1 (0.2)
Arousal (0 to 4)	1.8 (0.7)	0.5 (0.1)	2.1 (0.5)
**Pictures**
Valence (-2 to 2)	1.0 (0.3)	0.1 (0.3)	-1.3 (0.3)
Arousal (0 to 4)	1.6 (0.6)	0.6 (0.2)	2.6 (0.4)
**Faces**
Valence (-2 to 2)	1.1 (0.3)	-0.1 (0.1)	-1.2 (0.2)
Arousal (0 to 4)	1.5 (0.2)	0.8 (0.1)	2.5 (0.4)


#### Valence

As expected, results showed a main effect of emotion category, *F*(2,207) = 1526.762, *p* < 0.001, $\eta_{p}^{2}$ = 0.937. Positive stimuli were rated as more pleasant than neutral and negative stimuli, *p*s < 0.001; likewise, negative stimuli received lower valence ratings than neutral stimuli, *p*s < 0.001. A main effect of stimulus domain, *F*(2,207) = 9.528, *p* < 0.001, $\eta_{p}^{2}$ = 0.084, revealed that words were overall rated as more pleasant than pictures and faces, *p*s < 0.01, whereas the latter domains did not differ from each other. Additionally, an interaction of emotion category and stimulus domain, *F*(4,207) = 3.94, $\eta_{p}^{2}$ = 0.071, was due to the fact that the differences between stimulus domains were limited to positive stimuli (where words received higher valence ratings than pictures, *p* < 0.05) and neutral stimuli (with higher valence ratings for words and pictures in comparison to faces, all *p*s < 0.050).

## DISCUSSION

The present study aimed to investigate emotion effects in the processing of words, pictures, and facial expressions. Emotion effects in form of EPN and LPC were visible in all stimulus domains. Interestingly, results furthermore showed valence-specific biases depending on stimulus domain: within the verbal domain, ERPs indicated a preferential processing of positive content, whereas a bias for negative valence became evident for both pictures and facial expressions. In disagreement with previous assumptions (Hinojosa et al., 2009), however, words did not receive lower arousal ratings than pictures or facial expressions.

The finding that emotion effects in EPN and LPC amplitudes occurred in all three stimulus domains is in line with previous studies reporting comparable emotion-related activity for emotional words and pictorial stimuli. First, Schacht and Sommer (2009a) reported EPN and LPC modulations for both emotional words and emotional facial expressions. In a second study, words elicited similar emotion-related activations in P300 amplitudes compared to pictograms and pronounced emotion effects in the LPC time window (Tempel et al., 2013). Finally, in an fMRI study by Schlochtermeier et al. (2013), emotional words and pictures elicited comparable emotion-related activity. Nonetheless, these and the present results seem to be at contrast with a number of studies suggesting reduced capability of words as compared to pictures or faces to elicit emotion effects in ERPs (e.g., Hinojosa et al., 2009). A possible explanation for this pattern of results was recently proposed by Rellecke et al. (2011), suggesting that emotional processing in faces was automated to a higher degree than for words: In a highly superficial face-word decision task, emotion effects beyond 100 ms after stimulus onset were limited to face stimuli, although the exact same words had elicited EPN and LPC effects in another study using a lexical decision task (Palazova et al., 2011). These findings thus point to the importance of taking differences in task-dependence between stimulus domains into account. Notably, studies reporting *comparable* emotion-related activity for words and faces/pictures employed tasks that required lexico-semantic processing of verbal stimuli like a lexical decision task (Schacht and Sommer, 2009a), a valence judgment task (Schlochtermeier et al., 2013; Tempel et al., 2013), or an occasional recognition memory task in the present study. In contrast, investigations reporting *reduced* emotion effects for words used superficial tasks that could be performed on the basis of coarse perceptual features without requiring lexico-semantic processing, e.g., a face-word decision task (Rellecke et al., 2011), or a discrimination of intact stimuli within a series of scrambled distractors (Hinojosa et al., 2009). In line with these findings, Frühholz et al. (2011) reported that EPN effects for emotional faces occurred for both implicit (color naming) and explicit (emotion judgment) tasks, whereas EPN modulations for emotional words were limited to the valence judgment task. Thus, it seems that emotional words do not generally show a reduced capability to elicit emotion effects, but it seems to be automated to a much lesser degree than for facial expressions and pictures. This suggestion was also corroborated by task comparisons within the verbal domain, which suggested a high task-dependence of emotion effects, especially for the LPC (Fischler and Bradley, 2006; Schacht and Sommer, 2009b), but – more recently – also for the EPN (Hinojosa et al., 2010; Bayer et al., 2012b).

Considering previous findings, the present study also points to the importance of context effects for emotion effects in word processing. In a study investigating the influence of font size on emotion processing (Bayer et al., 2012a), the same stimulus words than in the present study did not receive a positive valence bias, but elicited EPN and LPC modulations for both positive and negative stimuli. Importantly, this difference occurred although the same task was employed in both studies. Furthermore, stimuli were presented in a blocked design and not intermixed with other stimuli (pictures and faces in the present study and words in large font size, respectively), a design that is likely to even reduce context effects. In case that (single) words are actually presented in direct context with “competing” stimuli, the impact on emotional processing in words seems to be even larger. This was shown for face stimuli (Rellecke et al., 2011), but also for linguistic context information (Bayer et al., 2010). Taken together, emotion effects for written words might not only depend on the immediate task at hand, but also on the broader experimental context as provided by previously presented stimuli.

Although emotion effects in ERPs were evident in all stimulus domains, results showed clear differences in valence biases between stimulus domains. For facial expressions, EPN and LPC effects were limited to negative stimuli. In the case of pictures, sensory processing of emotional content as evidenced by the EPN was visible for negative compared to neutral pictures, and, with a later onset, also for positive compared to neutral pictures. In the later LPC interval, however, emotion effects were largest for negative stimuli. In contrast, emotion effects were limited to positive stimuli in the verbal domain. Furthermore, words overall received more positive ratings than pictures or facial expressions. These results are in line with a bias for positive valence for words (e.g., Herbert et al., 2009; Bayer et al., 2012b) and a negative valence bias for pictures (e.g., Carretié et al., 2001) and facial expressions (e.g., Pourtois and Vuilleumier, 2006) as evidenced in previous reports. Interestingly, analyses of the anterior positivity in the EPN time window only partly corresponded to EPN effects. In the verbal domain, enhanced ERP amplitudes to positive compared with neutral words were in accordance with both the EPN as well as with previous literature on anterior (P2) effects of emotional content (Kanske and Kotz, 2007). In contrast, discrepancies were more pronounced for facial expressions. Here, both positive and negative faces differed from neutral faces at anterior electrode sites, while EPN effects were limited to negative faces, both in comparison to neutral and positive faces. Enhanced frontocentral positivities were previously reported in a similar time window (155–200 ms) by Eimer and Holmes (2002) for fearful compared to neutral facial expressions; comparisons, however, are limited by the fact that this study did not include positive stimuli. Concerning the EPN in response to emotional facial expressions, the time course of the component is of special interest since it temporally coincides with the N170 component. Although the dissociation between emotion effects in the EPN and the N170 has been a matter of debate, recent research suggested the involvement of at least partially dissociable neural generators (Rellecke et al., 2011, 2013). Lastly, anterior emotion effects were absent for pictures. Taken together, these results suggest that anterior effects are not merely counterparts of the posterior EPN effects in the same time window, suggesting a domain-specific involvement of multiple neural sources already at early stages of emotion processing.

On a theoretical level, the positive valence bias for words was related to a so-called positivity offset, describing the notion of a preference for positive stimuli at rather low levels of emotional activation, which was suggested to be the basis of approach motivation in neutral contexts (Cacioppo and Gardner, 1999). In contrast, results for pictures and faces are in line with the idea of a negativity bias for stimuli at higher arousal levels, which was supposed to prepare the organism for rapid responses to threatening or dangerous stimuli (see Cacioppo and Gardner, 1999). The assumption of generally lower arousal of words in comparison to pictorial stimuli is well comprehensible considering the arbitrary nature of written language, which requires the translation of symbols into meaningful concepts. Interestingly, however, this assumption was not corroborated by arousal ratings collected in addition to the present ERP study, where words did not receive reduced arousal ratings in comparison to pictures or facial expressions. In our opinion, this finding warrants careful interpretation. Instead of assuming that words do actually hold the same potential to elicit arousal reactions as pictures or facial expressions, we tentatively suggest that arousal ratings for words might reflect different aspects of the arousal concept than in the case of pictorial stimuli. Arousal ratings for words might thus – to a higher degree than in the case of pictorial stimuli – reflect a mainly cognitive evaluation of an underlying concept (cf., Bayer et al., 2011). In contrast, pictures hold much more imminent information (e.g., about possible dangers emanating from a stimulus) and might thus enable a more realistic assessment of its actual arousal value, i.e., the potential of a stimulus to elicit arousal reactions, by accounting for a bodily aspect of arousal. Further evidence for this assumption arises from the present data when relating emotion effects in ERPs to arousal ratings within stimulus domains. In the case of pictures and facial expressions, arousal ratings accurately mirror emotion effects in ERPs, with higher arousal ratings for negative pictures or facial expressions as compared to neutral or positive stimuli. For words, however, there is less agreement between ratings and ERP effects. Although a clear bias for positive valence was evident in ERPs, arousal ratings revealed no significant differences between negative and positive words, and even showed numerically larger values for the negative stimulus category. Undoubtedly, future research will need to corroborate the assumption of systematic differences in arousal ratings between pictorial and symbolic stimuli, and should elucidate whether these assumed differences are influenced by the presentation mode of stimuli, that is whether they are presented block-wise (as in the present study) or fully randomized. Furthermore, it would require developing instruments able to capture different aspects of the arousal concepts, which then might also shed new light on ambiguous findings concerning the role of stimulus arousal in previous research.

As discussed above, emotional processing within words, pictures, and facial expressions exhibits a number of notable differences concerning valence processing and task dependence. When considering these differences and their possible causes, it is unfeasible to neglect dissimilarities between stimulus domains themselves. As already mentioned, a major *processing* difference between written words and pictorial stimuli concerns the symbolic nature of the former stimulus class. In ERPs, this difference is reflected in EPN onsets, which were located at around 170–190 ms for pictures and facial expressions, but only started at 250 ms for words, reflecting the increased time necessary to gain access to lexico-semantic information. Above that, stimuli of these domains differ notably in their basic physical features. At this point, it seems noteworthy to make a distinction between basic domain-specific physical features and emotion-specific features. Concerning the former, written language is usually comprised of highly similar symbols without variability in size or color. Likewise, faces (of a given ethnicity) exhibit a highly distinctive arrangement of features with rather small differences between individuals. In contrast, pictures of objects or scenes show a high variability concerning visual complexity, colorfulness, or scope. Since stimuli were presented in a naturalistic form in the present study and thus differed in size, color, and complexity between domains, these differences became obvious in GFP activations averaged over emotion conditions within each domain, where pictures elicited by far the largest activations. Given these fundamental differences in basic activations between stimulus domains, the present study avoided any analyses of emotion by domain interactions.

Apart from general physical differences, it is interesting to consider the level at which the distinction between emotional and neutral stimuli becomes manifest: Within written words and pictures, these distinctions are presumably related to the emotional *meaning* (given careful stimulus selection). In contrast, within facial expressions, distinctions between neutral and emotional expressions are *determined* by specific arrangements of facial features (as, for example, described by facial action units) and thus completely depend on differences in physical properties. Furthermore, as stated in the introduction, it was discussed that facial expressions of emotion comprise only indirect affective information (Walla and Panksepp, 2013), most likely depicting an emotional reaction toward a direct affective stimulus. As a consequence, facial expressions of emotion are usually classified using the concept of basic emotions, while pictures and words are most often described via two-dimensional constructs comprising valence and arousal. For these reasons, it seems impossible to realize an experimental design with fully matching semantic information across domains – while one can include a picture of a cat as well as the word “cat,” it is impossible to select a matching facial expression^2^. Taken together, these points illustrate that domain-specific differences in physical properties and processing requirements should be taken into account when interpreting similarities and differences between emotion effects in specific stimulus domains.

In summary, the present study compared emotion effects in ERPs elicited by words, pictures, and facial expressions. In order to maximize their comparability, stimuli were presented in within-subject design using a task that ensured attentive processing with mostly identical demands on perceptual and cognitive resources across domains. Results showed that emotion effects in form of EPN and LPC occurred in all stimulus domains, but revealed pronounced differences in valence processing between stimulus domains. While emotion effects were limited to positive stimuli in the verbal domain, they were predominant for negative pictures and faces. In addition, words received generally higher valence ratings than pictures and facial expressions. Interestingly, assumed differences in arousal level between stimulus domains were not reflected in arousal ratings collected in the present study, possibly due to the involvement of different evaluative aspects in these ratings. Taken together, the present results point toward systematic differences in the processing of written words and pictures or facial expressions and thus advise caution in the interpretation and comparison of both results as well as underlying concepts across stimulus domains.

## Conflict of Interest Statement

The authors declare that the research was conducted in the absence of any commercial or financial relationships that could be construed as a potential conflict of interest.

## Footnotes

1 Data for words and pictures were collected in separate experiments from different participant samples.

2 In the present study, we approached this problem by focusing solely on angry facial expressions, accepting that this would result in reduced emotional variability in comparison to negative pictures and words, since they were not pre-selected in this regard. On the other hand, angry facial expressions were supposed to be particularly well suited for activating the human fear system (see Schupp et al., 2004b), and were thus frequently used in previous research (e.g., Schupp et al., 2004b; Rellecke et al., 2011, 2012). Above that, recent research suggested that differences in EPN and LPC between (negative) emotion categories in face processing seem to be negligible (Recio et al., 2014).
